# Left atrium remodeling predicts late recurrence of paroxysmal atrial fibrillation after second generation cryoballoon ablation

**DOI:** 10.1186/s12947-018-0137-8

**Published:** 2018-09-25

**Authors:** Andreea Motoc, Juan-Pablo Abugattas, Bram Roosens, Esther Scheirlynck, Benedicte Heyndrickx, Carlo de Asmundis, Gian-Battista Chierchia, Steven Droogmans, Bernard Cosyns

**Affiliations:** 1 Centrum Voor Hart-en Vaatziekten (CHVZ), Department of Cardiology, UZ Brussel, Laarbeeklaan 101, 1090 Brussels, Belgium; 2 Heart Rhythm Management Centre, UZ Brussel, Laarbeeklaan 101, 1090 Brussels, Belgium

**Keywords:** Atrial fibrillation, Cryoballoon ablation, Left atrium, Echocardiography

## Abstract

**Background:**

Atrial fibrillation (AF) is the most common arrhythmia worldwide. Nowadays, AF ablation is a valuable treatment option. It has been shown that the left atrium (LA) diameter is a predictor of AF recurrence after cryoballoon ablation (CBA). Since it does not reflect the true LA size, we compared the role of different LA anatomical parameters using echocardiography for the prediction of AF recurrence after CBA.

**Methods:**

We retrospectively included 209 patients (mean age 56.1 ± 13.6 years, male 62%) with paroxysmal AF undergoing CBA. A transthoracic echocardiography was performed in all patients.

**Results:**

At a mean follow-up of 16.9 ± 6.3 months, AF recurred in 25.4% of the patients. LA anterior - posterior diameter (LAD), LA minimum volume (LAmin) and early AF recurrence were independent predictors of recurrence. Based on receiver operating characteristics, cut – off values for LAD and, LAmin were 41 mm, 23.69 mL, respectively. The negative predictive values for recurrence were 73% and 87.3% respectively. In patients with AF recurrence, a significant proportion (30.2%) showed LA longitudinal remodeling (LA superior – inferior diameter) even though classically measured LAD was normal.

**Conclusions:**

Longitudinal LA remodeling plays an additional role for predicting AF recurrence after CBA, in patients without LAD dilation. Moreover, LAmin had a high negative predictive value and was an independent predictor of AF recurrence. Therefore, a more complete LA anatomical assessment allows a better prediction of AF recurrences after CBA.

## Background

Atrial fibrillation (AF) is the most common cardiac arrhythmia, with an increasing frequency worldwide, and it is associated with an elevated risk for stroke, heart failure and mortality. Its prevalence in developed countries is currently estimated to 1.5–2% of the general population [1]. Resinusalisation is one of the main goals in AF patients. Several ablation strategies have proven to be efficient in the treatment of AF [2, 3]. Among them, one of the most promising and effective approaches is the second generation cryoballoon ablation (CBA) (Arctic Front Advance, Medtronic). However, up to 20% of patients experience AF recurrence after CBA [4, 5].

Recent data have shown that an increase of the left atrium (LA) anterior - posterior diameter (LAD) assessed by echocardiography is a powerful predictor of recurrence after CBA [4, 6]. A possible explanation for the recurrence of AF could be the presence of myocardial fibrosis and remodeling of the LA [7]. However, LAD does not reflect the true size of the LA [8, 9]. We hypothesized that a more complete analysis of the LA dimensions could be a better predictor of AF recurrence after CBA. Therefore, we compared the role of different anatomical parameters of the LA measured by echocardiography for predicting the recurrence of AF after CBA.

## Methods

### Patient characteristics

We retrospectively included 209 patients having undergone CBA for paroxysmal AF in our center from January 2014 to February 2016.

Baseline demographic characteristics can be found in Table 1. CHA_2_DS_2_-VASc score was calculated according to European Society of Cardiology Guidelines [1].

**Table 1:** Baseline clinical and demographic characteristics

Characteristic	Study population (*n* = 209)	AF recurrence – (*n* = 156)	AF recurrence + (*n* = 53)	*P* value
Age (y)	56.1 ± 13.5	55.6 ± 13.5	57.4 ± 13.7	0.335
Male gender (*n*, %)	130 (62.2)	98(62.8)	32 (60.4)	0.753
Body mass index (kg/m^2^)	26.5 ± 4.7	26.3 ± 4.5	27 ± 5.3	0.549
Tobacco use (*n*, %)	52 (24.8)	35 (28.8)	17(32)	0.281
Hypertension (*n*, %)	86 (41.1)	59 (37.8)	27 (50.9)	0.094
Diabetes (*n*, %)	16 (7.7)	9 (5.8)	7 (13.2)	0.079
Dyslipidemia (*n*, %)	75 (35.9)	55 (35.3)	20 (37.7)	0.747
History of heart failure (*n*, %)	6 (2.9)	4 (2.6)	2 (3.8)	0.651
Coronary artery disease (*n*, %)	15 (7.2)	10 (6.4)	5 (9.4)	0.464
History of TIA^a^/CVA^b^(*n*, %)	19 (9.1)	15 (9.6)	4 (7.5)	0.653
Failed drugs before ablation				
Flecainide (*n*, %)	46 (22)	31(19.8)	15(28.3)	0.177
Propafenone(*n*, %)	1(0.47)	0 (0)	1(1.8)	0.083
Amiodarone(*n*, %)	9 (4.3)	6 (3.8)	3 (5.6)	0.555
Sotalol (*n*, %)	24 (11.4)	16 (10.2)	8 (15)	0.316
Metoprolol (*n*, %)	2 (0.9)	2 (1.2)	0 (0)	0.412
Bisoprolol (*n*, %)	78 (37.3)	57 (36.5)	21 (39.6)	0.620
Nebivolol (*n*, %)	6 (3.8)	5 (3.2)	1 (1.8)	0.632
ACE^c^ inhibitors before ablation	26 (12.4)	20 (12.8)	6 (11.3)	0.809
ARBs^d^ before ablation	11 (5.2)	9 (5.7)	2 (3.7)	0.592
Prior ablation	39 (18.6)	29 (18.5)	10 (18.8)	0.547
- AVNRT^e^	13 (6.2)	12 (7.7)	1 (1.9)	1.000
- Right atrial flutter	13 (6.2)	8 (5.1)	5 (9.4)	1.000
- AF^f^	11 (5.3)	8 (5.1)	3 (5.7)	1.000
- Other	2 (1.0)	1 (0.6)	1 (1.9)	1.000
CHA_2_DS_2−_VAsc score	1.3 ± 1.4	1.2 ± 1.3	1.6 ± 1.7	0.134
Oral anticoagulation (*n*, %)	60 (28.7)	41 (26.3%)	19 (35.8%)	0.552
Aspirin (*n*, %)	23 (11)	17 (10.9)	6 (11.3)	0.874
Follow-up duration (months)	16.8 ± 6.3	16.9 ± 5.7	16.6 ± 7.7	0.795
Re-do ablation	31 (14.8)	0 (0)	31 (58.4)	–
Medication in BP^g^				
- Flecainide (*n*, %)	62 (29.6)	42 (26.9)	20 (37.7)	0.122
- Propafenone (*n*, %)	2 (0.95)	1 (0.64)	1 (1.8)	0.415
- Amiodarone (*n*, %)	9 (4.3)	6 (3.8)	3 (5.6)	0.561
- Sotalol (*n*, %)	49 (23.4)	34 (21.8)	15 (28.3)	0.310
- Metoprolol (*n*, %)	1(0.4)	1(0.6)	0 (0)	0.562
- Bisoprolol (*n*, %)	111 (53.11)	81 (51.9)	30 (56.6)	0.497
- Nebivolol (*n*, %)	4 (1.9)	4 (2.5)	0 (0)	0.241
- ACE inhibitors (*n*, %)	27 (12.9)	21 (13.4)	6 (11.3)	0.710
- ARBs (*n*, %)	6 (2.8)	6	0	0.150
Medication after BP				
- Flecainide (*n*, %)	25 (11.9)	17 (10.8)	8 (15)	0.398
- Propafenone (*n*, %)	2 (0.9)	0 (0)	2 (3.7)	0.060
- Amiodarone (*n*, %)	6 (2.8)	3 (1.9)	3 (5.6)	0.154
- Sotalol (*n*, %)	26 (12.4)	18 (11.5)	8 (15)	0.478
- Metoprolol (*n*, %)	1 (0.4)	1(0.6)	0 (0)	0.562
- Bisoprolol (*n*, %)	62 (29.6)	50 (23.9)	12 (22.6)	0.211
- Nebivolol (*n*, %)	6 (2.8)	5 (3.2)	1 (1.8)	0.628
- ACE inhibitors (*n*, %)	27 (12.9)	21 (13.4)	6 (11.3)	0.710
- ARBs (*n*, %)	6 (2.8)	6	0	0.150
Recurrence in BP	8 (3.8)	0 (0)	8(15.1)	< 0.001

^a^*TIA* Transient ischaemic attack, ^b^*CVA* Cerebrovascular accident, ^c^*ACE* inhibitors, angiontensin – converter enzyme inhibitors, ^d^ *ARBs* angiotensin receptor blockers, ^e^ *AVNRT* atrioventricular nodal reentry tachycardia, ^f^*AF* atrial fibrillation, ^g^*BP* blanking period

The study was approved by the local Ethical Committee and was carried out in accordance with the ethical principles for medical research involving human subjects established by Helsinki’s Declaration, protecting the privacy of all participants, as well as the confidentiality of their personal information. All patients provided written informed consents.

### Transthoracic echocardiography

**Fig. 1:**
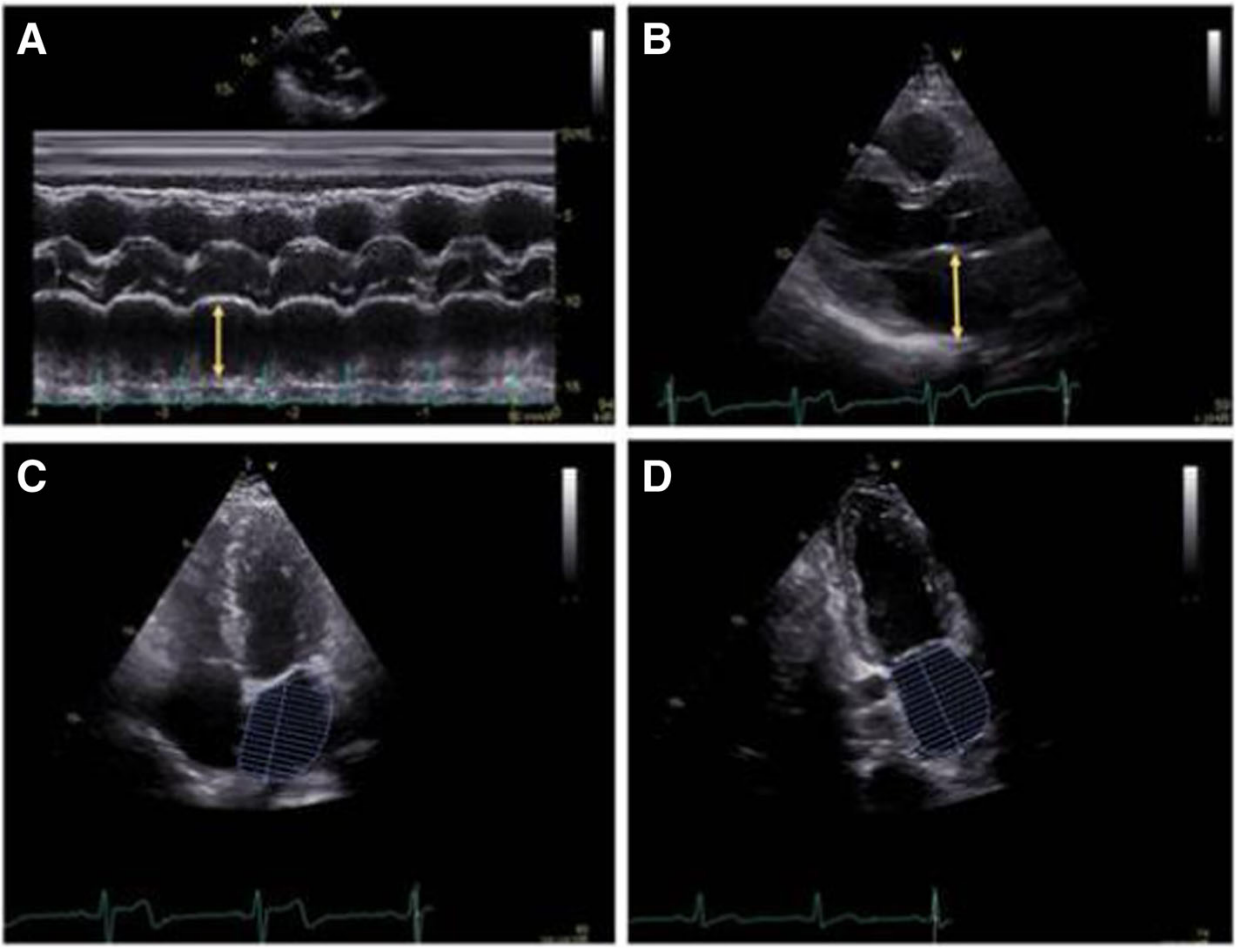
**a.** Illustration of the left atrium anterior – posterior diameter measurement in M-Mode, using the parasternal long axis view. **b.** Left atrium anterior – posterior diameter measurement in 2D parasternal long axis view. **c.** Left atrium volume measurement in four – chamber view. **d.** Left atrium volume measurement in two – chamber view

**Fig. 2:**
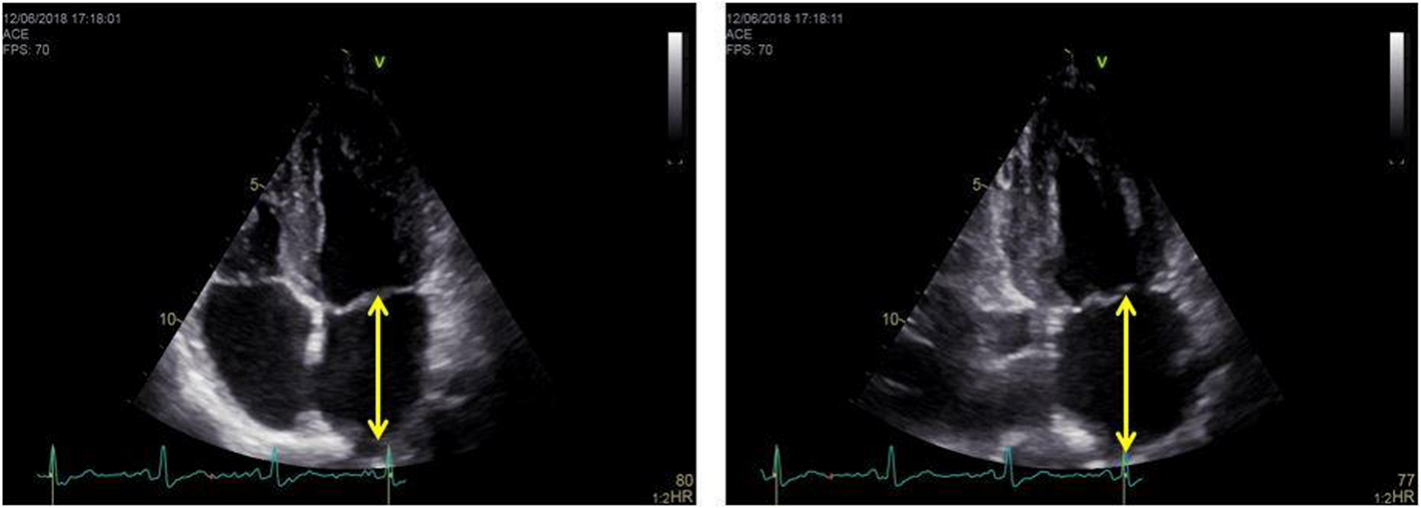
Left atrium length (superior - inferior diameter) is measured perpendicular from the mid-point of the segment that unifies the hinge points of the mitral leaflets to the roof of the left atrium

A comprehensive transthoracic echocardiography (TTE) (using GE Vingmed Ultrasound, Vivid E9, Horten, Norway; Phillips Epiq, Philips, Andover, Massachusetts) was performed in all patients, according to the recommendations [10, 11]. Standard parasternal long and short axis views and apical two-, three - and four - chamber views were available in all patients. Left ventricular (LV) dimensions were measured using M – Mode in the parasternal long axis view. LAD was measured using anatomical M-mode and two – dimensional assessment (2D) in the parasternal long axis view. LA maximum (LAmax) and minimum (LAmin) volumes were measured in two - and four chamber views, using the biplane area-length method (Fig. 1). LA volumes were indexed based on the patient’s body surface area. Left atrium superior - inferior diameter was measured in apical two - and four chamber views (Fig. 2). Doppler mitral inflow peak early diastolic (E), peak atrial systolic velocities (A) and deceleration time were measured. Tissue Doppler imaging at the level of the mitral annulus was used to measure peak early diastolic septal and lateral e’, as well as peak late diastolic a’, corresponding to the P-wave on the electrocardiogram (ECG) [10, 12].

To determine the reproducibility of the LAD, LA superior – inferior diameter, LAmax and LAmin, the measurements were repeated in 20 randomly selected patients by an additional investigator and by the same primary investigator 1 week later. During the repeated analysis, the investigators were blinded to the results of all previous measurements.

### Pre-procedural management

The antiarrhythmic drugs (AADs) were discontinued at least 3 days before the procedure, except for amiodarone, which was stopped one month before.

### Cryoballon ablation procedure

Our standard ablation procedure has been previously reported in detail [13]. Briefly, after obtaining LA access, through a steerable 15 Fr sheath (FlexCath Advance®, Medtronic©), an inner lumen mapping catheter (ILMC) (Achieve®, Medtronic©) was advanced in each pulmonary vein (PV) ostium and baseline electrical information was gathered. Optimal vessel occlusion was considered as achieved upon selective contrast injection showing total contrast retention with no backflow into the atrium. Once occlusion was documented, cryothermal energy was started for at least 180 s. Usually, the PVs were treated as follows: first, the left superior PV (LSPV), then the left inferior (LIPV), right inferior (RIPV) and right superior (RSPV). PV activity was recorded with the ILMC at a proximal site within the ostium prior to ablation in each vein. If PV potentials (PVPs) were visible during energy delivery, time to isolation was recorded as the time from the start of the cryoenergy application until the PVPs completely disappeared or were dissociated from LA activity. In cases of phrenic nerve palsy (PNP), recovery of diaphragmatic contraction was carefully monitored for 30 min. Further additional cryoenergy applications were not applied if the veins were isolated after the initial freeze. If needed, pacing from the distal and/or proximal coronary sinus was performed to recognise far field atrial signals from PVPs recorded on the mapping catheter. During the whole procedure, activated clotting time was maintained > 250 s. In order to avoid phrenic nerve palsy, diaphragmatic stimulation was achieved by pacing the phrenic nerve during septal pulmonary veins ablations.

### Postablation management

Patients were discharged the day after ablation if the clinical status was stable. Following the procedure, all the patients were continuously monitored with ECG telemetry for at least 18 h. Oral anticoagulation was not discontinued for the ablation and continued for at least 2 months following the CHA_2_DS_2_ - VAsc score. During the blanking period (BP), AADs were continued. The decision to continue AADs after the BP or to perform a repeat procedure was taken if a first episode of recurrence of AF occurred.

### Follow-up

All the included patients underwent physical examination and a 24 h Holter recording at 1, 3, 6 and 12 months after the ablation. Additional Holter monitoring was performed if arrhythmic symptoms occurred. All documented AF episodes > 30 s were considered as a recurrence. A BP of 3 months was considered for the study.

### Statistical analysis

Continuous variables are expressed as mean ± standard deviation. Categorical variables are expressed as percentages. Comparisons of continuous variables were done with a Student t-test or Mann-Whitney U-test and binomial variables with a chi-square or Fisher test as appropriate. Receiver-operator characteristic (ROC) curves were constructed to evaluate the performance of variables in predicting AF recurrence and to calculate adjusted cut-off values, specificity and sensitivity of the parameters (using Youden’s Index) in the prediction of AF recurrence. Freedom from AF recurrence was estimated by Kaplan-Meier method and time-dependent comparisons by the log-rank test. In order to evaluate potential predictors of AF recurrence, a separate Cox proportional hazard model was used. Intraclass correlation coefficient (ICC) was used to determine the intra- and inter - observer variability. The calculated ICCs were judged as follows: 0.50 to 0.74 poor to moderate, 0.75 to 0.92 good and > 0.93 excellent. Statistical significance was considered with a *P*-value < 0.05. Statistical analyses were conducted using IBM SPSS Statistic for Windows, Version 24.0 (Armonk, NY: IBM Corp.)

## Results

At a mean follow – up of 16.8 ± 6.3 months after the BP, 53 (25.4%) patients presented recurrences of AF.

### Baseline population characteristics and recurrences

Baseline clinical and demographic characteristics of the study population are listed in Table 1. There was a significant difference between the groups with and without recurrence for patients who presented early AF recurrence.

Echocardiographic characteristics are presented in Table 2. In brief, there was a significant difference in patients with and without recurrence respectively for LV end-diastolic diameter (50.3 ± 7.9 mm vs. 47.2 ± 6.5 mm, *p* = 0.013), LV mass index (83.7 ± 28.4 g/m^2^ vs. 76.6 ± 26.3 g/m^2^, *p* = 0.045), LAD (42.2 ± 7.2 mm vs. 39 ± 6.1 mm, *p* = 0.013), LAmax (67.3 ± 27.8 mL vs. 57.7 ± 21.2 mL, *p* = 0.036), LAmin (36.7 ± 20.1 mL vs. 27.5 ± 14.9 mL, *p* = 0.004) and LAmin indexed (18.1 ± 9.3 mL/m^2^ vs. 14.1 ± 7.4 mL/m^2^, *p* = 0.007).

**Table 2:** Echocardiographic parameters

Characteristic	Study population (*n* = 209)	AF recurrence – (*n* = 156)	AF recurrence + (*n* = 53)	*P* value
Heart rate during echocardiography	75.6 ± 14.5	76.3 ± 14.1	75.6 ± 14.5	0.080
Left ventricle				
- LVEF^a^, %	54.4 ± 5.2	54.6 ± 4.7	53.8 ± 6.4	0.343
- LV^b^ EDD^c^, mm	48 ± 7	47.2 ± 6.5	50.3 ± 7.9	0.013
- LV ESD^d^, mm	30.4 ± 5.8	30.4 ± 5.9	30.5 ± 5.7	0.939
- IVS_d_^e^, mm	9.5 ± 1.7	9.5 ± 1.78	9.6 ± 1.7	0.763
- PW_d_^f^, mm	9.3 ± 1.6	9.1 ± 1.4	9.6 ± 1.8	0.131
- LV mass, g	140.4 ± 72.8	136.8 ± 70.1	150.9 ± 80	0.073
- LV mass index, g/m^2^	78.4 ± 26.9	76.6 ± 26.3	83.7 ± 28.4	0.045
- LV EDV^g^, mL	99 ± 32.5	96.4 ± 30.5	109.8 ± 39.6	0.223
- LV ESV^h^, mL	46.8 ± 20.8	45.4 ± 20.6	52.4 ± 21.6	0.196
Left atrium				
- LA^i^ antero-posterior diameter, mm	39.8 ± 6.5	39 ± 6.1	42.2 ± 7.2	0.013
- LA antero-posterior diameter indexed, mm/m^2^	20.4 ± 3.4	20.1 ± 3.3	20.4 ± 3.4	0.182
- LA superior- inferior diameter, mm	48.8 ± 7.6	48.3 ± 7.6	50.3 ± 7.4	0.095
- LA maximum volume, mL	60 ± 23.3	57.7 ± 21.2	67.3 ± 27.8	0.036
- LA maximum volume indexed, ml/m^2^	30.4 ± 10.8	29.5 ± 10	33.5 ± 12.6	0.067
- LA minimum volume, mL	29.8 ± 16.8	27.5 ± 14.9	36.7 ± 20.1	0.004
- LA minimum volume indexed, mL/m^2^	15.1 ± 8	14.1 ± 7.4	18.1 ± 9.3	0.007
Doppler				
- E- wave velocity, m/s	0.6 ± 0.1	0.6 ± 0.1	0.6 ± 0.1	0.768
- A – wave velocity, m/s	0.5 ± 0.1	0.5 ± 0.1	0.4 ± 0.1	0.405
- E/A (ratio)	1.4 ± 0.5	1.3 ± 0.5	1.4 ± 0.5	0.314
- DTE^j^, ms	180.8 ± 44.4	180.2 ± 45.4	182.7 ± 41.6	0.482
- TDI^k^ e’ septal, cm/s	7.4 ± 2.1	7.3 ± 2	7.8 ± 2.5	0.366
- TDI e’ lateral,cm/s	9.4 ± 4.9	9.6 ± 5.3	9 ± 2.8	0.965
- E/e’ avg. (ratio)	8.6 ± 2.9	8.7 ± 2.9	8.4 ± 3.2	0.893
- TDI a’ septal, cm/s	7.4 ± 2	7.6 ± 2	6.8 ± 2	0.053
- TDI a’ lateral, cm/s	8 ± 2.4	8.1 ± 2.4	7.5 ± 2.4	0.111
Mitral regurgitation (*n*, %)	97 (46.4)	70 (44.8)	27 (50.9)	0.594
- Mild (*n*, %)	90 (43.1)	64 (41)	26 (49.1)	
- Moderate (*n*, %)	7 (3.3)	6 (3.8)	1 (1.9)	
Mitral annulus calcification (*n*, %)	10 (4.8)	7 (4.5)	3 (5.7)	0.677
Aortic regurgitation (mild) (*n*, %)	24 (11.9)	20 (12.8)	4 (7.5)	0.298
Tricupid regurgitation (*n*, %)	106 (50.7)	77 (49.3)	29 (54.7)	0.338
- Mild (*n*, %)	96 (45.9)	70 (44.8)	26 (49)	
- Moderate (*n*, %)	9 (4.3)	7 (4.4)	2 (3.7)	
- Severe (*n*, %)	1 (0.4)	0 (0)	1 (1.8)	
TAPSE^l^, mm	23.1 ± 4	23 ± 3.7	23.5 ± 4.9	0.253
IVC^m^, mm	14.3 ± 4	14.1 ± 3.9	15.1 ± 4.2	0.258
TR^n^ gradient, mmHg	24.9 ± 7.5	25.1 ± 7.7	24.1 ± 6.9	0.783

^a^*LVEF* left ventricle ejection fraction, ^b^*LV* left ventricle, ^c^*EDD* end – diastolic diameter, ^d^*ESD* end – systolic diameter, ^e^*IVSd* Interventricular septum end-diastolic diameter, ^f^*PWd* posterior wall end-diastolic diameter, ^g^*EDV* end – diastolic volume, ^h^*ESV* end – systolic volume, ^i^*LA* left atrium, ^j^*DTE* deceleration time of E – wave, ^k^*TDI*, tissue Doppler imaging, ^l^*TAPSE* tricuspid annulus plane systolic excursion, ^m^*IVC* inferior vena cava, ^n^*TR* tricuspid regurgitation

### Procedural data

All the patients underwent the procedure with the large 28-mm CBA. At the beginning of the procedure, 203 (97.1%) patients were in sinus rhythm. The mean total procedure and fluoroscopy time were 66.9 ± 20.2 min, respectively 13.8 ± 7.5 min. The mean number of freeze-thaw cycles was 1.2 ± 0.4 in the LSPV, 1.1 ± 0.3 in the LIPV, 1.19 ± 0.44 in the RSPV, 1.2 ± 0.5 in the RIPV. The mean minimal temperatures obtained were − 48.7 ± 8.6 °C in LSPV, − 48.7 ± 8.6 in LIPV, − 49.2 ± 10.8 in RSPV, − 46 ± 14.6 in RIPV. There were no significant differences regarding the procedural data between the two groups. Procedural data can be found in Table 3.

**Table 3:** Procedural data

Characteristic	Study population (*n* = 209)	AF recurrence – (*n* = 156)	AF recurrence + (*n* = 53)	*P* value
Procedural time, min	66.9 ± 20.2	67 ± 20.3	66.5 ± 19.9	0.918
Fluoroscopy time, min	13.8 ± 7.5	13.4 ± 7.2	14.7 ± 8.3	0.452
Freezes in LSPV^a^	1.2 ± 0.4	1.2 ± 0.4	1.2 ± 0.4	0.656
Freezes in LIPV^b^	1.1 ± 0.3	1.1 ± 0.4	1.1 ± 0.3	0.215
Freezes in RSPV^c^	1.1 ± 0.4	1.1 ± 0.4	1.2 ± 0.4	0.086
Freezes in RIPV^d^	1.2 ± 0.5	1.2 ± 0.4	1.2 ± 0.5	0.366
LSPV freeze duration, s	223.6 ± 78.9	224.8 ± 80.5	220 ± 74.6	0.782
LIPV freeze duration, s	208.2 ± 65.5	211.1 ± 69	199.7 ± 53. 7	0.412
RSPV freeze duration, s	208.5 ± 79	206 ± 75	215.9 ± 89.8	0.647
RIPV freeze duration, s	225.4 ± 89.1	223 ± 89.4	232.5 ± 88.8	0.147
Min temp^e^ in LSPV, °C	−48.7 ± 8.6	−48.7 ± 9.5	−48.6 ± 5	0.325
Min temp in LIPV, °C	−45.4 ± 8.6	- 45.5 ± 9.5	− 44.9 ± 5.4	0.192
Min temp in RSPV, °C	−49.2 ± 10.8	−49.3 ± 11.9	−47 ± 14.6	0.246
Min temp in RIPV, °C	−46 ± 14.6	− 45.4 ± 16.3	−47.9 ± 6.5	0.698
Pulmonary veins variants	102 (48.8)	79 (50.6)	23 (43.3)	0.347
- Left common ostium	58 (27.8)	42 (26.9)	16 (30.2)	0.647
- Right middle pulmonary vein	29 (13.9)	25 (16.0)	4 (7.5)	0.124
- Right common ostium	8 (3.8)	7 (4.5)	1 (1.9)	0.640
- Other	7 (3.3)	5 (3.2)	2 (3.8)	0.686

^a^*LSPV* left superior pulmonary vein, ^b^*LIPV* left inferior pulmonary vein, ^c^*RSPV* right superior pulmonary vein, ^d^*RIPV* right inferior pulmonary vein, ^e^Min temp, minimum temperature

### Global versus longitudinal LA remodeling and AF recurrences

**Fig. 3:**
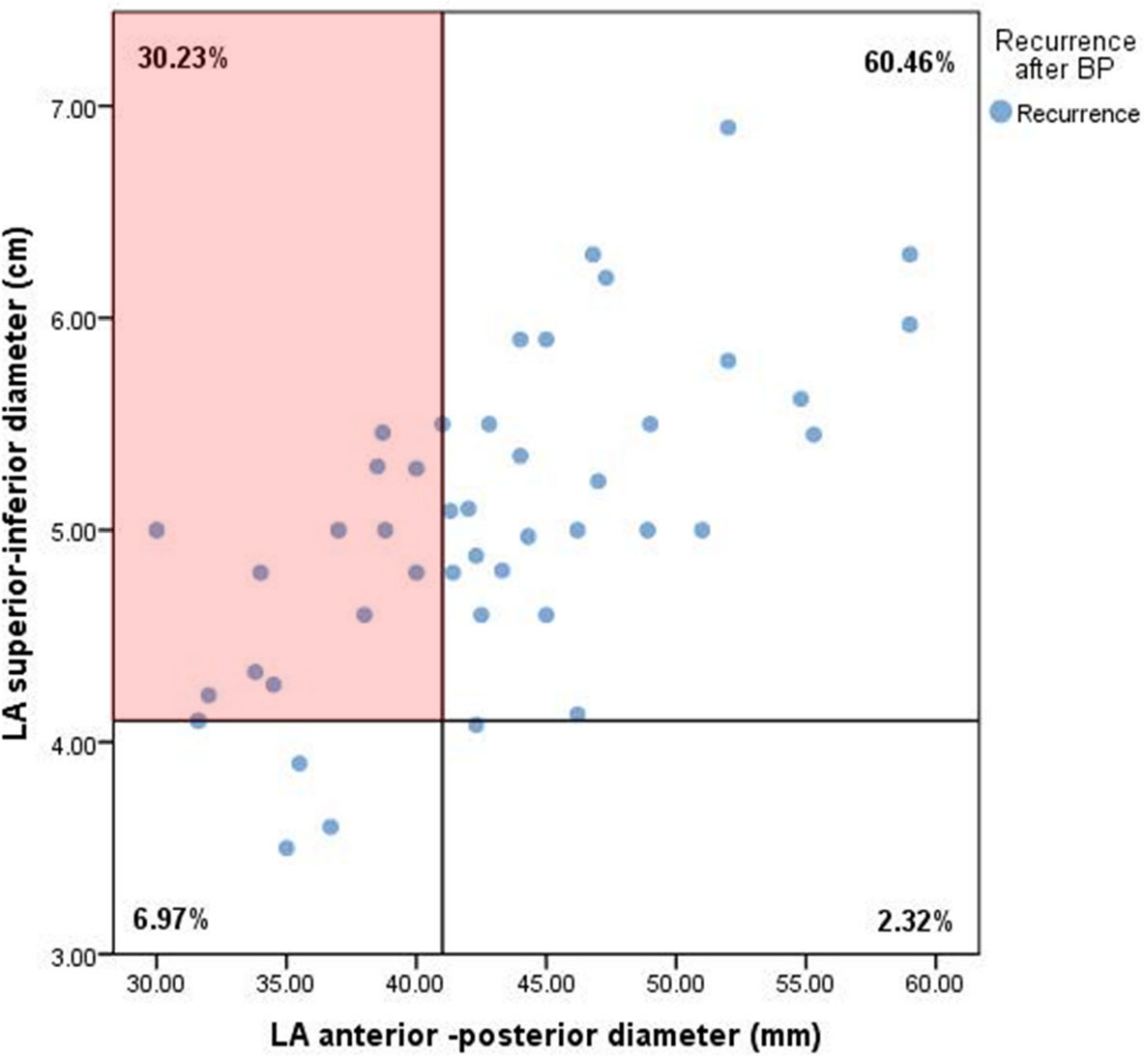
Proportions of patients with AF recurrences according to the adjusted cut – off values for LA superior – inferior diameter (41 mm) and LA anterior – posterior diameter (41 mm), showing that 30.2% of patients with recurrence had longitudinal remodeling, in the absence of an increased anterior-posterior diameter

Fig. 3 illustrates that using only the LAD, with an adjusted cut-off value of 41 mm, led missing up to 30.2% of AF recurrences. In the patients with recurrence without LAD dilation, a significant number of patients had longitudinal LA remodeling (increased superior – inferior LA diameter, with an adjusted cut-off value of 41 mm).

### LA volumes and AF recurrences

Multivariate regression analysis showed that LAD, LAmin and early AF recurrence were independent predictors of AF recurrence after the BP, as illustrated in Table 4.

**Table 4:** Predictors of atrial fibrillation recurrence

Parameters			Multivariate analysis					
	Univariate analysis		Model 1		Model 2		Model 3	
	HR^h^ (95% CI^i^)	*p*	HR (95% CI)	*p*	HR (95% CI)	*p*	HR (95% CI)	*p*
AHT^a^	1.608 (0.937–2.762)	0.085	1.675 (0.886 - 3.166)	0.112	1.489 (0.771–2.875)	0.236	1.433 (0.773–2.658)	0.254
DM^b^	2.141 (0.963–4.763)	0.062	1.447 (0.549–3.816)	0.455	1.410 (0.541–3.679)	0.482	1.389 (0.523–3.688)	0.509
Recurrence in BP^c^	4.867 (2.174–10.892)	< 0.001*	4.457 (1.647–12.062)	0.003*	4.990 (1.884–13.215)	0.001*	4.504 (1.830–11.088)	0.001*
LV^d^ mass index	1.010 (0.998–1.022)	0.100	1.008 (0.993–1.023)	0.301	1.007 (0.992–1.021)	0.366	1.003 (0.990–1.015)	0.681
LAD^e^	1.017 (1.006–1.028)	0.003*	1.062 (1.010–1.117)	0.018*				
LAmax^f^	1.024 (1.011–1.036)	< 0.001*			1.009 (0.996–1.022)	0.157		
LAmin^g^	1.078 (1.031–1.127)	0.001*					1.016 (1.001–1.031)	0.039*

^a^*AHT* arterial hypertension, ^b^*DM* diabetes mellitus, ^c^*BP* blanking period, ^d^*LV* left ventricle, ^e^*LAD* left atrium diameter, ^f^*LAmax* left atirum maximum volume, ^g^*LAmin* left atrium minimum volume, ^h^*HR* hazard ratio; ^i^*CI* confidence interval, *p < 0.05

ROC analysis revealed that for a cut-off value of 23.69 ml for LAmin it was associated with a high sensitivity for predicting AF recurrence after the BP (79.5% sensitivity, 46% specificity). For the above mentioned adjusted cut-off values, LAmin had a high negative predictive values for AF recurrences 87.3% .

In contrast, for LAD, sensitivity and specificity were 56.3% and 66%, respectively, with a lower negative predictive value of 73% compared to LAmin.

### Reproducibility

Intraobserver variability was excellent for all the parameters. For LAD the ICC was 0.972, for LA superior – inferior diameter ICC = 0.95, for LAmax ICC = 0.964 and for LAmin ICC = 0.940. Interobserver reliability was good for LAD (ICC = 0.906), LAmax (ICC = 0.907) and LAmin (0.860) and excellent for LA superior – inferior diameter (ICC = 0.96).

## Discussion

The main findings of our study are: 1) recurrence of AF after the BP occurred in 25.4% of all subjects with paroxysmal AF. 2) In the group of patients with AF recurrence, longitudinal remodeling (increased superior – inferior LA diameter) was also present in patients with normal LAD. 3) In the global population of patients undergoing CBA, LA minimum volume was an independent predictor of AF recurrences after the BP. These results suggest that AF recurrence after ablation also occurs in the absence of global LA remodeling assessed by LAD.

This study evaluates the role of additional anatomical parameters of the LA using two-dimensional echocardiography for the prediction of recurrence of AF after the BP in patients who have undergone CBA.

The incidence of recurrence of AF after the BP in our study was of 25.4%, which is consistent with results of previous studies. Gerede et al. [6] have reported a rate of recurrence after the BP of 31.3%. In a recent study published by Coutino et al. [5] they showed a rate of recurrence after the BP of 25.2%. Previous studies have shown that the presence of recurrence during the BP is an independent predictor of late AF recurrence, which was also confirmed by our analysis [14, 15].

Previous studies have shown that the enlargement of LAD is an independent predictor of recurrence of AF after the BP, suggesting that the remodeling of the LA is a leading cause for late recurrences of AF [4, 6]. This is consistent with our results that report LAD as a predictor of recurrence of AF after CBA.

However, the remodeling of the LA can occur non-uniformly, correlating with reduced success of different ablation techniques [9, 16] and furthermore, LAD is not considered as representative of LA dimension following the EACVI/ASE recommendations [10, 11] . Our study shows that 30.2% of the patients with a normal LAD, but a dilated superior – inferior diameter presented AF recurrence after the BP. This novel finding suggests that longitudinal remodeling is also involved in AF recurrence after ablation. However, longitudinal LA dilation could also be observed in patients without recurrence and was not significantly predictive for AF recurrence. These data shows that LA remodeling is a spatial process.

Therefore, LA volumes assessment may be a more discriminative parameter of remodeling to predict AF recurrence.

Interestingly, our study showed that LAmin was an independent predictors of AF recurrence after the BP, and that it was associated with a high sensitivity for predicting recurrence for the adjusted cut-off values (79.5%).

Limited data is available regarding the value of LA minimum volume and its prognostic value for different cardiovascular events. LAmin volume was associated with the occurrence of AF in an elderly cohort, as showed by Fatema et al. [17] and it was the most accurate parameter reflecting anatomical remodeling associated with paroxysmal AF in a recent study performed by Schaaf et al. [7]. In several studies, LA minimum volume was associated with diastolic dysfunction starting from incipient stages [18, 19]. However, we did not find a significant difference regarding the diastolic function between the groups with and without recurrence. These findings suggest that LA minimum volume represents a more subtle form of LA remodeling that could also trigger the occurrence of AF recurrence after ablation.

Whether LA minimum volume may have a better prognostic value in predicting late AF recurrences following cryoballoon ablation requires further investigations.

As suggested by Canpolat et al. [20], CBA may have a positive impact on LA reverse remodeling. Since the remodeling is a continuous process, measurements performed at a single moment may not reflect the truth and a temporal trend of the LA volumes should be assessed. However, data regarding LA reverse remodeling is limited and there are no clear definitions for it, therefore it should be evaluated in further studies.

### Study limitations

This is a single center retrospective study, therefore results may not be generalized. Larger prospective clinical studies are warranted to confirm our findings. Moreover, the present study addressed the prognostic value of LA anatomy only in paroxysmal AF and cannot be extrapolated to a persistent AF population undergoing CBA.

### Future perspectives

As mentioned earlier, it is widely accepted that anatomical remodeling is associated with recurrence of AF after ablation [4, 6, 9, 16, 21, 22]. 2D echocardiography can provide useful information for the detection of patients at risk to develop recurrences of AF, but LA volumes are still based on only two orthogonal planes and related to geometrical assumptions. Our results suggest that LA longitudinal remodeling could play an additional role in the prediction of AF recurrences, as the expansion of the LA can be asymmetrical. Therefore, advanced imaging techniques such as three – dimensional (3D) echocardiography can overcome these limitations and provide more accurate and reproducible LA measurements [23]. Moreover, several studies showed that LA strain could predict recurrence of AF after catheter ablation. Therefore, strain imaging may bring additional information regarding the more subtle structural and functional left atrium remodeling in such patients and could be used for the follow-up assessment of possible reverse atrial remodeling [24–26].

## Conclusions

The results of our study showed that LA longitudinal remodeling, assessed by the increase of LA superior – inferior diameter plays an additional role in AF recurrence after CBA. Moreover, LA minimum volume was an independent predictors of AF recurrences after the BP, suggesting that less advanced forms of LA remodeling, not detected by the dilation of LAD can trigger AF recurrences. Further large – scale prospective randomized studies are needed to confirm our results.

## Abbreviations

AADs: Antiarrhythmic drugs
AF: Atrial fibrillation
BP: Blanking period
CBA: Cryoballoon ablation
ECG: Electrocardiogram
ICC: Intraclass correlation coefficient
ILMC: Inner lumen mapping catheter
LA: Left atrium
LAD: Left atrium anterior - posterior diameter
LAmax: Left atrium maximum volume
LAmin: Left atrium minimum volume
LIPV: Left inferior pulmonary vein
LSPV: Left superior pulmonary vein
LV: Left ventricle
PNP: Phrenic nerve palsy
PV: Pulmonary vein
PVPs: Pulmonary vein potentials
RIPV: Right inferior pulmonary vein
ROC: Receiver - operator characteristic
RSPV: Right superior pulmonary vein
TTE: Transthoracic echocardiography
